# Cervical Ectopic Pregnancy Presenting With Ruptured Posterior Cervical Lip: A Case Report

**DOI:** 10.7759/cureus.28508

**Published:** 2022-08-28

**Authors:** Felix A Elachi, Kenneth Egwuda, Christopher O Egbodo, Deborah O Olubiyi

**Affiliations:** 1 Obstetrics and Gynaecology, Alps Hospitals and Diagnostics, Jos, NGA; 2 Reproductive Biology, Alps Hospitals and Diagnostics, Jos, NGA; 3 Obstetrics and Gynaecology, Jos University Teaching Hospital, Jos, NGA; 4 Family Medicine, Alps Hospitals and Dignostics, Jos, NGA

**Keywords:** cervical repair, vasopressin, incomplete miscarriage, ruptured cervical lip, cervical ectopic gestation

## Abstract

Cervical ectopic pregnancy (CEP) is a rare but fatal early pregnancy complication. A rare case of cervical ectopic gestation with rupture of the posterior cervical lip is reported due to the rarity of this presentation and the need to consider it in patients presenting with miscarriages. We present a 19-year-old G_2_P_0_^+1^ at a gestational age of 11 weeks and six days who presented with a cervical ectopic gestation with rupture of the posterior cervical lip. Transvaginal ultrasound showed features of a cervical ectopic gestation. The products of conception were evacuated through the cervical defect posteriorly after infiltration of vasopressin and the defect on the posterior lip was repaired. A ruptured cervical lip is a possible presentation of CEP. It may present the clinician with a diagnostic challenge. A high index of suspicion and proficiency in transvaginal ultrasonography is required for prompt diagnosis.

## Introduction

Ectopic gestation, a life-threatening gynaecological emergency is an important cause of early pregnancy morbidity and mortality particularly in the tropics [1]. It is said to occur when a pregnancy implants outside the endometrial lining of the uterine cavity [1]. In cervical ectopic pregnancy (CEP) the blastocyst implants in the cervical canal [2].

A retrospective review of all ectopic pregnancies in London over a seven-year revealed a CEP rate of one in 150 ectopic pregnancies [3]. A nine-year review in southern Nigeria revealed that there were 2.2 ectopic pregnancies for every 100 deliveries; however, no case of CEP was reported [4]. In other parts of the world, CEP constitutes less than 1% of ectopic gestations with incidence reported to range between one in 1,000-18,000 pregnancies [5]. This infrequent presentation of CEP has made epidemiological studies difficult [6]. Also, there are no studies comparing the clinical features with a controlled group [6].

The exact cause of CEP has not been established [6]. A systematic review of the literature posited that most risk factors were related to factors that lead to injuries to the cervix which facilitated ectopic implantation in the cervix [6]. Also stated as a risk factor were conditions that resulted in endometrial injuries which prevent normal eutopic implantation [6].

CEP may be a diagnostic dilemma [7]. It may be mistaken for the cervical phase of incomplete abortion of a normally sited pregnancy, cervical fibroid and cancer of the cervix [6]. Early diagnosis and treatment are life-saving and preserve fertility in CEP [7]. A high index of suspicion is recommended because of its severe outcome if the diagnosis is delayed [8]. The utilization of ultrasonographic diagnostic criteria has increased the early diagnosis of CEP allowing for early intervention [9]. We report this case of CEP with rupture of the posterior cervical lip, which was managed at Alps Hospitals and Diagnostics, Jos, to highlight the rarity of this presentation and the need to consider it in patients presenting with miscarriage.

## Case presentation

The patient was a 19-year-old student of the Berom ethnic group of Plateau State, Nigeria. She was G2P0+1 and her gestational age was 11 weeks and six days on presentation. She presented to the emergency unit of our facility with complaints of abdominal pain with vaginal bleeding of two days duration. The abdominal pain was located in the suprapubic region and was colicky. She had vaginal spotting of blood, the blood was altered and contained neither vesicles nor fleshy materials. She noticed the above symptoms after oral ingestion of four tablets of a brand of prostaglandin E1 analogue for the termination of pregnancy two days prior to presentation. She had no vaginal instrumentation at any time before the presentation. She had no history of dizziness or collapse.

The index pregnancy was unwanted. She decided to terminate the pregnancy as she was not ready to have a child and also the person that got her pregnant was not supportive. Her first pregnancy was two years ago. She had dilatation and curettage for pregnancy termination at a gestational age of about eight weeks. She did not have any post-abortal complications.

She was not a known hypertensive or diabetic. She has not had any surgical intervention in the past. She had no known drug allergies. She was studying Television programming. She was the first child in a monogamous family of six children. Both of her parents were farmers. She neither drank alcohol nor used tobacco in any form.

Examination revealed a young lady that was anxious. She was not febrile with a temperature of 36.8^o^C and was not pale. Her pulse was 90 beats per minute, it was regular and had a full volume; the blood pressure was 130/90mmHg. Her respiratory rate was 18 cycles per minute and the breath sounds were vesicular. Her abdomen was full and moved with respiration, there were no areas of tenderness and no palpable organs nor masses. Pelvic examination revealed a vulva and vagina that were stained with blood, there was a pool of blood in the posterior vaginal fornix. The cervix was bulky with an open os. A cystic mass was noted distending the cervical os.

A diagnosis of inevitable miscarriage at 11 weeks and six days was made. She was informed of the diagnosis and line of further management. Intravenous access was secured with a size 18G cannula and blood samples were obtained for investigations. The serum pregnancy test was positive, her packed cell volume was 35%, and her blood group was B rhesus positive. She was screened for retroviral disease and hepatitis B and C and she was non-reactive. An abdominal ultrasound done revealed ‘a bulky uterus with a mixed echogenic collection distending the cervical canal, there were no adnexal masses and no fluid collection in the pelvis. Impression - cervical phase of incomplete abortion’.

**Figure 1:**
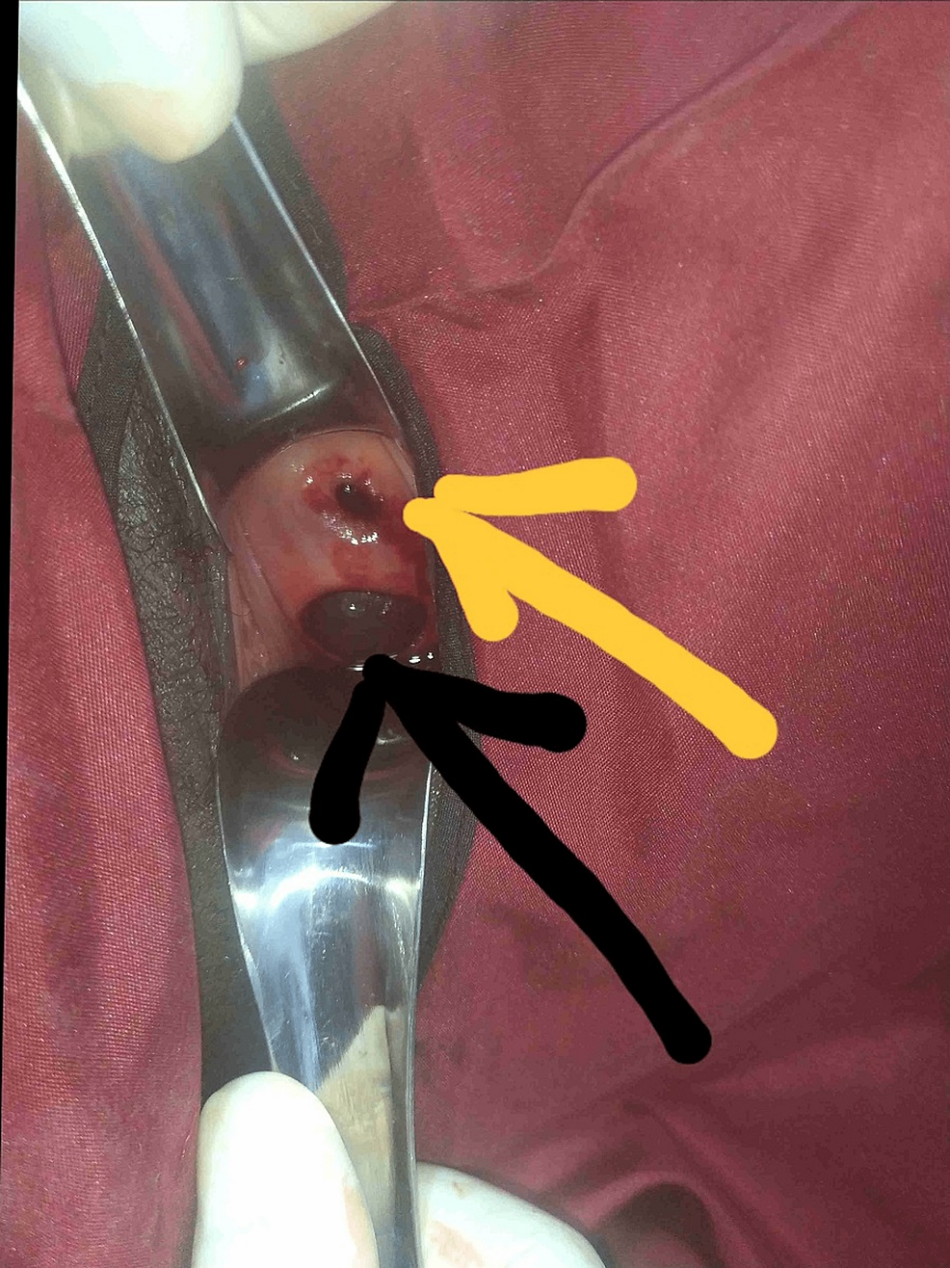
Exposed cervix with a yellow arrow pointing to the external os and the black arrow pointing to rent in the posterior lip of cervix.

She was informed of the need for a manual vacuum aspiration (MVA) of products of conception. She consented to the procedure and was taken to the procedure room for MVA under sedation. Pelvic examination was repeated by the Medical Officer under sedation. The cervix was bulky and the os was open. A 4cm defect was noted in the posterior lip of the cervix with a cystic mass protruding through the defect (Figure 1). The attention of the Consultant Gynaecologist was drawn and she was reviewed. History and examination findings were essentially as earlier noted above. An impression of CEP with ruptured posterior cervical lip was made.

**Figure 2:**
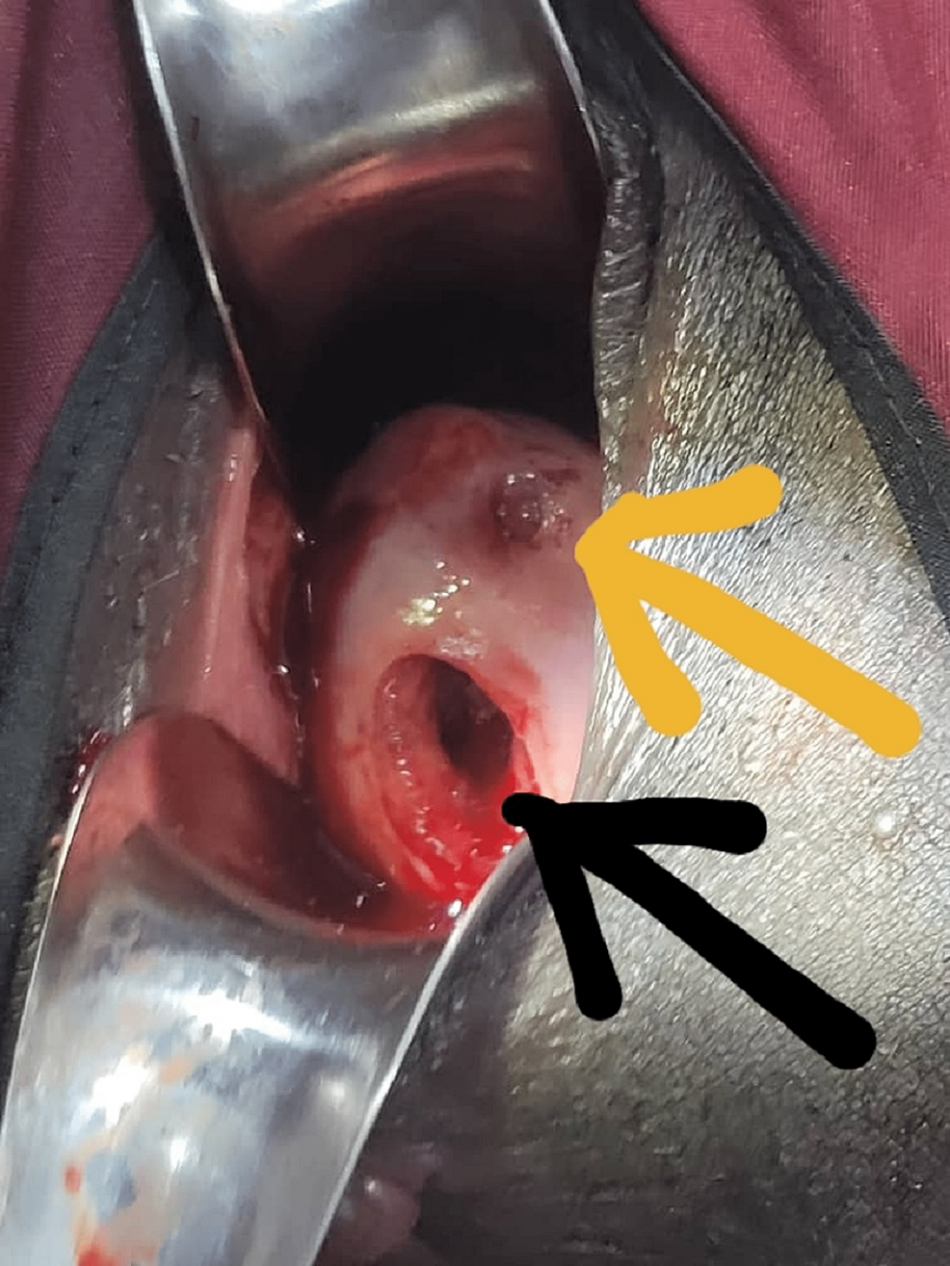
The exposed cervix after removal of products of conception. Yellow arrow points to the external cervical os and the black arrow to the rent on the posterior lip.

A transvaginal ultrasound revealed an empty uterus and a bulky cervix that gave an appearance of an ‘hour glass’. The cervix contains a cystic mass with echogenic content. The internal cervical os above the cystic mass was closed. The right ovary harbours a 2 by 2cm cystic mass. There was no fluid collection in their pouch of Douglas. An impression of a cervical ectopic gestation with a right corpus luteum cyst was made. This impression was explained to the patient and her mother. She was informed of the need for examination in theatre, cervical evacuation with trachelorrhaphy with a possible hysterectomy if bleeding becomes intractable. She then signed consent for the operation. She was then taken to the theatre for the above procedure under general anaesthesia. Intraoperative findings were essentially as earlier documented. A dilute solution of vasopressin was injected into the cervix taking care not to puncture the gestational sac and the gestational sac was delivered through the posterior cervical rupture. Figure 2 reveals the cervix after the removal of the products of conception.

**Figure 3:**
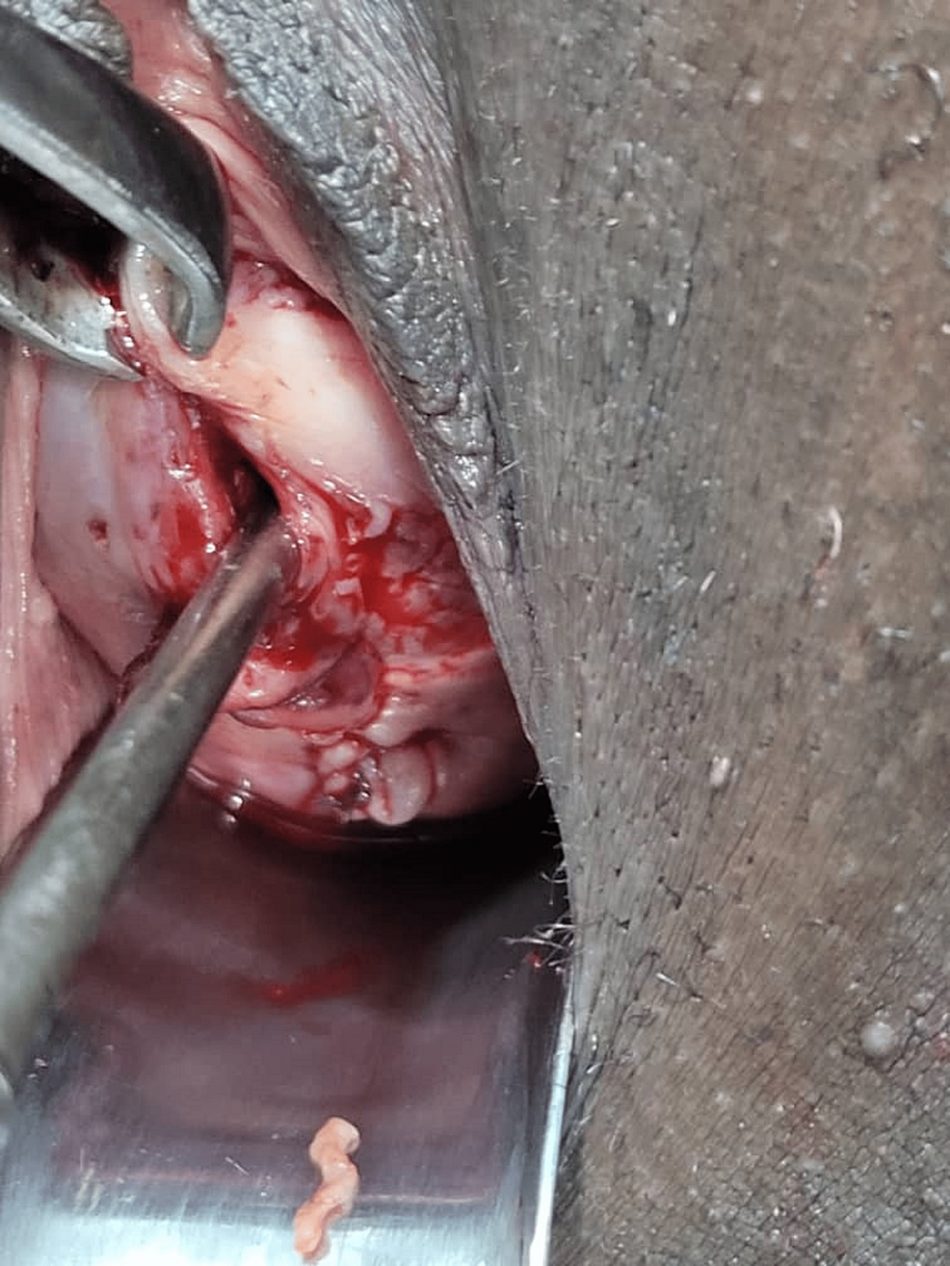
Exposed cervix with uterine sound in the os after repair

A uterine sound was inserted into the uterine cavity through the external cervical os to avoid occluding the cervical canal and the posterior cervical defect was repaired continuously with vicyl number 1 (Figure 3).

A check MVA of the uterine cavity was done and there was no significant aspirate. A size 8 Foley catheter was passed into the uterus and retained for 1 week to maintain the cervical canal. She had postoperative antibiotics (ciprofloxacin and metronidazole) and analgesics (diclofenac and pentazocin). The products of conception were sent for histology. She had an uneventful postoperative period. She did not have further vaginal bleeding and her vital signs were normal. Her postoperative packed cell volume 24 hours later was 32%. She was informed of the need for contraception. She was informed of danger signs such as profuse vaginal bleeding and to seek help immediately. She was discharged home. She was seen at the outpatient clinic on postoperative day 8. She did not have any complaints. Histological examination revealed the presence of chorionic villi and trophoblastic cells with cervical stroma and glands, and an impression of cervical pregnancy was made. Her general condition was satisfactory. Vaginal examination revealed a serous discharge in the posterior fornix and the wound on the posterior cervical lip was adjudged to be healing well. The intrauterine Foley catheter was removed. She was given a two-week appointment at the outpatient clinic but she defaulted. Several attempts were made to contact her on her telephone line but she could not be reached.

## Discussion

CEP is a rare and important complication of pregnancy due to its association with adverse outcomes [9,10]. Suboptimal health-seeking attitudes and delayed diagnosis are some factors responsible for the adverse outcomes in low and middle-income countries [10]. The rarity of CEP has hampered epidemiological studies, as a result, studies comparing its clinical features with a control group are rare [6].

Taskin et al reported a cervical intramural ectopic gestation with rupture of the anterior cervical lip [11]. We however did not come across any case report or series from our country or sub-region on CEP with a ruptured cervix. Just like in our patient with a previous dilatation and curettage, analysis from a systematic review revealed that the most important risk factor for CEP was related to a previous injury to the cervical canal from miscarriages, curettage or cervical surgery [6]. This is thought to facilitate cervical implantation through changes in cervical vascularisation and histologic changes [6]. These changes are also thought to also impair the competence of the internal cervical os resulting in leakage of embryos from the endometrial cavity [6]. It should be noted that absolute risk factors remain few due to their rarity, as a result, it is difficult to get epidemiological studies comparing the clinical presentation with a controlled group [12].

CEP is an important gynaecological condition due to its rarity and high propensity for massive haemorrhage and the need for extirpative surgeries [10,12]. A high index of suspicion and proficiency in ultrasonography are required for prompt diagnosis of CEP [10]. When a prompt diagnosis of ectopic gestation is made significant morbidity or mortality can be prevented [13]. Diagnosis of a cervical ectopic gestation may be missed if the clinician or radiologist is not conscious of the entity [13]. Cases of misdiagnosed CEP are continually been reported [12,13]. This was the case in this patient who presented with vaginal bleeds and abdominal pains after ingestion of misoprostol for termination of an unwanted pregnancy. There was no history of any intervention that could damage the cervix. The clinical features and features on abdominal ultrasonography were thought to be that of a cervical phase of an incomplete abortion; however, a review and transvaginal ultrasonography by a gynaecologist revealed features of CEP with ruptured posterior cervical lip. Transvaginal ultrasonography is the mainstay of diagnosis of CEP and this permits early diagnosis [14]. The clinical criteria for diagnosis of CEP include uterine bleeding without abdominal pains following a period of amenorrhoea, a soft and enlarged cervix equal to or larger than the uterus, products of conception confined and attached to the endocervix, a closed internal os and partially opened external os [15]. Most of these criteria were present in this patient. Early CEP can be mistaken for an incomplete or inevitable miscarriage because the product of conception is retained in the cervical canal by an unyielding external cervical os. The sliding sign however distinguishes this effectively, by this when gentle pressure is applied to the cervix with the probe an implanted CEP does not slide unlike the gestational sac of a miscarriage [16]. Also, a CEP gives high peritrophoblastic vascularity on doppler assessment helps to differentiate CEP from incomplete miscarriage [17].

The presentation of this patient with a rent in the posterior lip of the cervix was a rare presentation. Cases of CEP with a ruptured cervix are scarce in our environment and sub-region. Even though she ingested prostaglandin E1 analogue (misoprostol) for the termination of the pregnancy we do not think it was responsible for the rupture of the posterior cervical lip. This is because misoprostol will lead to ripening or softening of the cervix and uterine contraction as a result there is no resistance in the cervix that will lead to its rupture, unlike hypertonic saline which only causes uterine contraction without cervical ripening leading to a possible cervical or uterine rupture [11]. A non-visible cervical tear may be a passage through which an early pregnancy may also implant in the substance of the cervix [11].

The option of treatment of CEP includes conservative methods which could be medical, surgical or both and radical surgery [14]. This patient had evacuation of the products of conception from the cervix after infiltration of a dilute solution of vasopressin and cervical repair. This was because she was not bleeding profusely and was haemodynamically stable. This option of treatment will also preserve her reproductive potential. Intracervical injection of vasopressin reduces the risk of haemorrhage during surgery [18]. Other modalities that could reduce the risk of haemorrhage include transvaginal ligation of the cervical branch of the uterine artery, and cervical cerclage [17]. Post-operative bleeding can be controlled with intracervical tamponade using Foley’s catheter, application of haemostatic sutures at the implantation site, and bilateral uterine and internal artery ligation [17]. Where haemorrhage is continuous and uncontrollable hysterectomy can be opted. In women who have completed child bearing hysterectomy may be considered an initial option [17]. Medical management can be done with systemic administration of methotrexate or local injection into the cervix under ultrasound guidance [8]. Where foetal cardiac activity was observed ultrasound guided potassium chloride administration into the gestational sac or foetal thorax until the cessation of cardiac activity before administration of methotrexate [8].

## Conclusions

In conclusion, a ruptured cervical lip is a possible presentation of CEP. It may present the clinician with a diagnostic challenge. A high index of suspicion especially in patients who have had dilatation and curettage for termination of pregnancy in the past and proficiency in transvaginal ultrasonography is required for prompt diagnosis. Local injection of vasoconstriction like vasopressin at the site of rupture and repair especially in haemodynamically stable patients may be considered a treatment option.
